# Type 1 Diabetes Hypoglycemia Prediction Algorithms: Systematic Review

**DOI:** 10.2196/34699

**Published:** 2022-07-21

**Authors:** Stella Tsichlaki, Lefteris Koumakis, Manolis Tsiknakis

**Affiliations:** 1 Department of Electrical & Computer Engineering Hellenic Mediterranean University Heraklion Greece; 2 Institute of Computer Science Foundation for Research and Technology-Hellas Heraklion Greece

**Keywords:** type 1 diabetes, hypoglycemia, predictive models, continuous glucose monitoring, heart rate variability, artificial intelligence

## Abstract

**Background:**

Diabetes is a chronic condition that necessitates regular monitoring and self-management of the patient’s blood glucose levels. People with type 1 diabetes (T1D) can live a productive life if they receive proper diabetes care. Nonetheless, a loose glycemic control might increase the risk of developing hypoglycemia. This incident can occur because of a variety of causes, such as taking additional doses of insulin, skipping meals, or overexercising. Mainly, the symptoms of hypoglycemia range from mild dysphoria to more severe conditions, if not detected in a timely manner.

**Objective:**

In this review, we aimed to report on innovative detection techniques and tactics for identifying and preventing hypoglycemic episodes, focusing on T1D.

**Methods:**

A systematic literature search following the PRISMA (Preferred Reporting Items for Systematic Reviews and Meta-Analyses) guidelines was performed focusing on the *PubMed*, *Google* *Scholar*, *IEEE* *Xplore*, and *ACM* Digital Library to find articles on technologies related to hypoglycemia detection in patients with T1D.

**Results:**

The presented approaches have been used or devised to enhance blood glucose monitoring and boost its efficacy in forecasting future glucose levels, which could aid the prediction of future episodes of hypoglycemia. We detected 19 predictive models for hypoglycemia, specifically on T1D, using a wide range of algorithmic methodologies, spanning from statistics (1.9/19, 10%) to machine learning (9.88/19, 52%) and deep learning (7.22/19, 38%). The algorithms used most were the Kalman filtering and classification models (support vector machine, k-nearest neighbors, and random forests). The performance of the predictive models was found to be satisfactory overall, reaching accuracies between 70% and 99%, which proves that such technologies are capable of facilitating the prediction of T1D hypoglycemia.

**Conclusions:**

It is evident that continuous glucose monitoring can improve glucose control in diabetes; however, predictive models for hypo- and hyperglycemia using only mainstream noninvasive sensors such as wristbands and smartwatches are foreseen to be the next step for mobile health in T1D. Prospective studies are required to demonstrate the value of such models in real-life mobile health interventions.

## Introduction

Diabetes is a recurrent condition that involves constant control and self-management of the patient’s blood glucose. Improper regulation of blood glucose levels in patients with type 1 diabetes (T1D) can lead to severe problems, such as kidney and heart failure, stroke, and blindness [1]. In contrast, through appropriate care for diabetes, a patient can live a prosperous life. Nevertheless, an overly strict glycemic control can raise the likelihood of developing hypoglycemia, a rapid decrease in blood glucose levels, which may lead to coma and potentially death if proper care is not taken immediately.

The concern of hypoglycemia is a barrier to successful hyperglycemic control, as it encourages insulin underdoing. Methods of reducing hypoglycemia occurrences include instruction and counseling to increase hypoglycemia recognition in time, as well as the development of predictive technological approaches that could reduce the occurrences of hypoglycemia. Blood glucose self-monitoring requires a blood sample to be collected on many occasions throughout the day. Currently, the use of continuous glucose monitoring (CGM) systems allows the collection of blood glucose level information in real time. In contrast, modern wearables can produce and analyze great amounts of data, which is the reason why modern technologies are frequently used in conjunction with these products to process and retrieve valuable information from the collected data. They also have several different monitoring capabilities, such as GPS, heart rate, electrocardiogram (ECG), and skin temperature, which are all important for the assessment of diabetes-related indicators [2]. Furthermore, several key indicators for the physical and mental health state of patients with T1D, such as blood glucose levels, calories, physical activity, and stress level, can be monitored by evaluating the data obtained from wearables. The main advantage of these devices is their ability to keep track of the patient’s daily routine in a continuous and discreet manner without affecting their normal everyday activities.

Artificial intelligence algorithms have been widely used to predict diabetes or as diagnostic tools, especially for type 2 diabetes [3]. Machine learning models have been used to predict the near future blood glucose levels and inform patients to take appropriate actions in advance to avoid a hypo- or hyperglycemic episode [4]. An accurate predictor could improve the quality of life of patients with T1D.

The aim of this paper was to review the emerging detection methods and approaches for the identification of hypoglycemia episodes. Specifically, we investigated the methods used or invented to improve blood glucose monitoring and increase its effectiveness to estimate future glucose levels; this could contribute to the prediction process of future episodes of hypoglycemia. Overall, these methods are highly valuable based on whether they can aid the prediction process, which is critical in avoiding a potentially dangerous hypoglycemic episode that could lead to major health consequences. Finally, we discuss prediction approaches aimed at the early identification and prevention of nocturnal hypoglycemia episodes, which could lead to “dead-in-bed” syndrome if not identified early. These approaches are categorized as mentioned previously, and their proposed techniques are discussed.

## Methods

### Article Identification

A systematic literature search following the PRISMA (Preferred Reporting Items for Systematic Reviews and Meta-Analyses) guidelines [5] was performed. For this research, we used *PubMed*, *Google* *Scholar*, *IEEE* *Xplore*, and *ACM* Digital Library to find articles about technologies related to hypoglycemia detection in patients with T1D. After exploring and combining many search terms to ensure having the broadest results, we used the following terms: “hypoglycemia,” “prediction,” “detection,” “continuous glucose monitoring,” “CGM,” “type 1 diabetes,” “T1D,” “HRV,” “heart rate variability,” “machine learning,” and “deep learning.”

### Inclusion and Exclusion Criteria

The search was performed in June 2021 and was restricted to articles from 2005 onward. In parallel, an alert was set to avoid missing articles. References of selected articles were analyzed to extract other related articles, and a complementary search in Google Scholar was used to find further information when necessary and complete the review with original works on each subtopic identified. All the authors deliberated and agreed on the inclusion and exclusion criteria. In case of disagreements, these were resolved through discussion among the authors to reach a consensus. In the first step of the screening process, journal articles and conference papers were deemed suitable for inclusion, whereas letters, correspondence, and review articles were excluded from this systematic review. Articles reporting on new glucose sensors that exhibit a linear detection range wide enough for blood or interstitial measurement were eligible. For prediction algorithms, the eligible articles had to report methods for glucose prediction and present details on the data sets used, methodology, and performance metrics. We included algorithms that predicted glucose values in a defined prediction horizon, as well as those that specifically predicted hypoglycemic events up to a maximum of 24 hours in the future. To be eligible, a study had to focus on hypoglycemia or include hypoglycemia prediction or detection techniques based on patient data. The patient group had to have T1D, whereas the trials had to have a control group. Studies that described the same methodology and technology as an already included study without significant distinction were excluded. We excluded trials that focused on the primary prevention of diabetes, those targeting gestational diabetes, those pertaining to a closed-loop or artificial pancreas system, and those that primarily focused on type 2 diabetes.

## Results

### Study Selection

**Figure 1:**
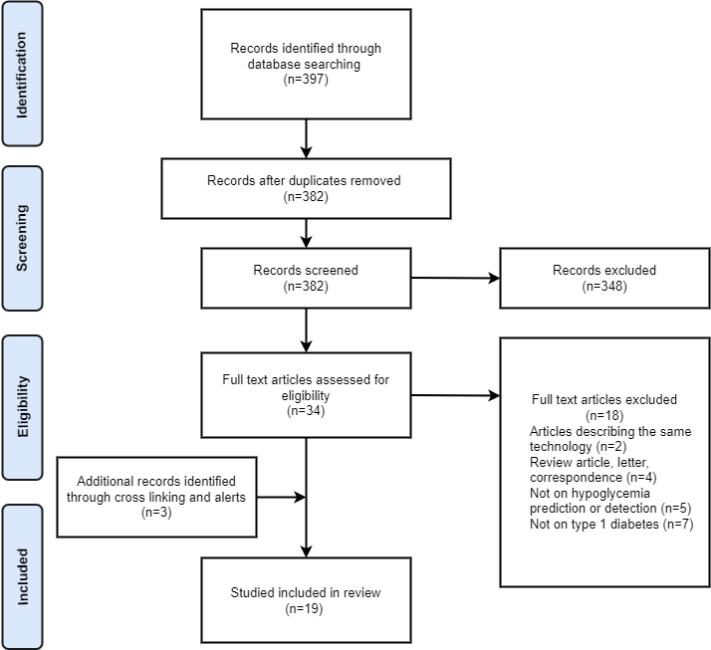
The PRISMA (Preferred Reporting Items for Systematic Reviews and Meta-Analyses) flow diagram presenting the search and screening strategy followed in this systematic review.

In total, the aforementioned literature search gave 397 results. Of the 397 records, 382 (96.2%) were screened after the removal of 15 (3.8%) duplicates, and 348 (87.7%) articles were excluded as they did not meet our eligibility criteria. After reading the full text of the remaining 34 articles, complimentary alerts helped to add 3 more articles that were also evaluated based on the aforementioned screening process, resulting in the inclusion of 19 eligible articles in total. Figure 1 presents the PRISMA flow diagram [5], illustrating the search and screening procedure of this review.

### Study Characteristics

Prediction algorithms aid in further enhancement of the quality of life of patients with T1D and their ability to avoid hypoglycemia. They enable patients to intervene early and successfully for the prevention of hypoglycemia episodes. Several of the approaches introduce novel algorithms for predicting hypoglycemia. However, only a few of them sought to assess their clinical efficacy and advantages in real-life settings. The details of each reviewed study are presented in Table 1, where we report the publication, the data set used, the technique on which the predictive model is based, and the resulting accuracy of the model.

**Table 1:** Summary of the reviewed hypoglycemia prediction approaches.

Study	Duration	Data set	Age (years)	Technique	Result
Mordvanyuk et al [6], 2017	500 simulated days	11 computer-generated adults through UVA-Padova T1D^a^ Simulator	>18	K-nearest neighbors	Accuracy 83.64%
Paul et al [7], 2015	6 weeks	6 patients from diabetes research in children network (DirecNet)	Mean 7 (SD 3)	Autoregressive models of higher and lower orders; state space model	Relative error (higher autoregressive) –7% Relative error (lower autoregressive) –24% Relative error (state space) –12%
Jensen et al [8], 2013	2 experimental sessions for each participant	10 male patients with T1D	Mean 44 (SD 15)	SVM^b^	AUC^c^-ROC^d^ 0.962 Sample-based sensitivity 81% Sample-based specificity 93% Event-based sensitivity 100%
Zhang et al [9], 2008	N/A^e^	Multiparameter Intelligent Monitoring in Intensive Care Database II	N/A	Classification tree	Accuracy 86% Sensitivity 89.87%
Dave et al [10], 2020	90 days	112 patients with T1D	Mean 11 (SD 10)	LR^f^ and RF^g^	Sensitivity (LR) 91.85% Specificity (LR) 96.25% Sensitivity (RF) 94.20% Specificity (RF) 96.67%
Eren-Oruklu et al [11], 2010	24 hours	54 patients with T1D	Mean 12.5 (SD 5.5)	Absolute predicted glucose values; cumulative sum; exponentially weighted moving average	Sensitivity 89%, 87.5%, and 89% Specificity 67%, 74%, and 78%
Chase et al [12], 2010	Overnight	40 patients with T1D	Mean 21 (SD 7.5)	Linear projection; Kalman filtering; hybrid infinite impulse; statistical prediction; numerical logical algorithm	Sensitivity 84%
Buckingham et al [13], 2013	21 nights	19 patients with T1D	≥18	Kalman Filtering	AUC algorithm 1 71% AUC algorithm 2 90% AUC algorithm 3 89%
Georga et al [14], 2013	From 5 to 22 days	15 patients with T1D	Mean 42 (SD 23)	Support vector for regression	Sensitivity (30-minute horizon) 92% Sensitivity (60-minute horizon) 96%
Bertachi et al [15], 2018	12 weeks	10 patients with T1D	>18	SVM	Sensitivity 78.75% Specificity 82.15%
Vahedi et al [16], 2018	4 months	93 patients with T1D	Mean 46 (SD 38)	MLP^h^ neural networks regressor	Mean absolute percentage error RF regressor 27.9% Mean absolute percentage error MLP regressor 29.6%
Maritsch et al [17], 2020	1 week	1 patient with T1D	N/A	Gradient boosting decision tree	Accuracy 82.7% Sensitivity 76.7% Specificity 84.2%
San et al [18], 2016	10 hours overnight	15 children with T1D	<18	Deep belief neural network and restricted Boltzmann machines	Sensitivity 80% Specificity 50%
Kuang et al [19], 2021	8 weeks	12 patients with T1D from the OhioT1DM data set	Mean 50 (SD 30)	Deep neural networks; LSTM^i^; artificial RNN^j^	30-minute prediction horizon (mg/dL) RMSE^k^ 19.10; MAE^l^ 13.59; glucose RMSE 22.08 60-minute prediction Horizon (mg/dL) RMSE 32.61; MAE 24.25; glucose RMSE 38.04
Zhu et al [20], 2020	360 days (simulation) and 8 weeks (clinical trial)	10 computer-generated adults through the UVA-Padova T1D Simulator and 6 patients with T1D from the OhioT1DM data set	Mean 49 (SD 31)	Dilated RNN and transfer learning	RMSE 20.1 mg/dL
Li, K, unpublished data, October 2019	6 months	10 computer-generated adults and 10 computer-generated children through the UVA-Padova T1D Simulator	>18 and <18	Deep reinforcement learning; double dilated RNN	Adults: glucose TIR^m^ 93% Children: glucose TIR 83%
Munoz-Organero et al [21], 2020	10 days (simulation) and 4 days (clinical trial)	40 computer-generated adults through the AIDA Diabetes software and 9 patients with T1D from the D1NAMO Open data set	N/A	LSTM and RNN	Computer-generated patients: RMSE <5 mg/dL Real patients: RMSE <10 mg/dL
Ranvier et al [22], 2016	5 days	1 patient with T1D	N/A	Decision tree	Model validation is in progress because of the lack of patient data variety
Cichosz et al [23], 2014	2 days	10 patients with T1D	Mean 44 (SD 15)	Forward selection and linear LR	Accuracy 99% Sensitivity 79%

^a^T1D: type 1 diabetes.

^b^SVM: support vector machine.

^c^AUC: area under the curve.

^d^ROC: receiver operating characteristic.

^e^N/A: not applicable.

^f^LR: logistic regression.

^g^RF: random forest.

^h^MLP: multilayer perceptron.

^i^LSTM: long short-term memory.

^j^RNN: recurrent neural network.

^k^RMSE: root mean square error.

^l^MAE: mean absolute error.

^m^TIR: time in target range.

### Hypoglycemia Prediction Algorithms

In a study by Mordvanyuk et al [6], authors examined 11 profiles of patients with T1D using the UVA-Padova T1D Simulator, which is a system developed at the Universities of Virginia and Padova, through research purposes. In their method, they presented the use of k-nearest neighbor on patient data, along with details relevant to a sequence of meals, to forecast a possible hypoglycemic or hyperglycemic episode. Their findings indicate that the use of consecutive data can dramatically improve the results of the prediction, especially when estimates determine the type of meal (ie, breakfast, snack, and lunch). Their approach obtained a sensitivity of 88% when taking into account only carbohydrate intake, fast-acting insulin dose, and premeal blood glucose.

In terms of blood glucose prediction, the algorithms used in these studies include linear autoregressive and state space time series models, classification algorithms such as the support vector machine (SVM), classification trees, logistic regression, and random forest [7-10]. Paul et al [7] studied the use of generalized autoregressive conditional heteroscedasticity (GARCHs) models on CGM profiles of young children with T1D. They aimed to analyze glucose time series and variability, as well as the feasibility of credible blood glucose level prediction. The forecasting capabilities of the GARCH methodology were compared with those of other existing modeling techniques, such as lower- and higher-order autoregressive models and state space models, where the GARCH method proved to be efficient in recognizing the variability of the glucose profiles and in providing a more credible prediction of short-term future blood glucose levels.

Our research was conducted specifically on patients with T1D, who have the greatest need for this type of prediction algorithm, as they are more complex because of their high sensitivity to exogenous factors and their increased blood glucose variability. In an experiment by Jensen et al [8], the authors established a pattern classification approach to enhance real-time hypoglycemia identification. They examined data from 10 patients with T1D, who experienced 17 insulin-induced hypoglycemic episodes. These episodes were then analyzed to extract characteristics, including the recent insulin intake time and the linear regression of the CGM signal, along with other measures (kurtosis and skewness), at different periods. The various combinations of features were used in an SVM model, and its performance was measured, resulting in the detection of all 17 hypoglycemic incidents, with 1 false positive and a lead time of 14 minutes.

Zhang et al [9] used a classification learning technique to forecast hypoglycemic events during a 1-hour time span. A classification tree was created using a data mining tool, and the input data comprised blood glucose measurements and insulin injection frequency. The accuracy and specificity of hypoglycemia prediction for the classification tree were 86% and 89%, respectively.

Dave et al [10] investigated 2 different approaches to effectively detect hypoglycemic episodes. These approaches comprised logistic regression and random forest. In their machine learning–based hypoglycemia detection method, they used data from 112 patients with T1D and relied on an extensive feature extraction process to identify any possible glucose patterns. Their final model was developed by considering linear and nonlinear models and combining the collected features. The proposed method correctly forecasted hypoglycemic episodes and achieved high sensitivities close to 95% and 94% and specificities of approximately 97% and 95% for prediction horizons of 0 to 15 and 15 to 30 minutes, respectively.

A few studies [11,12] incorporated different algorithms to improve the performance of their models and take advantage of the unique qualities of each algorithm. The different algorithms used in the included approaches were grouped based on their similarity and are presented in Multimedia Appendix 1.

Eren-Oruklu et al [11] examined 3 different time series–based methodologies for hypoglycemia forecasting on a data set of 54 patients with T1D. Their approach involved an exponentially weighted moving average and cumulative sum control chart, as well as the absolute values of the forecasted blood glucose levels. Each patient was fitted with a Medtronic CGM device that obtained blood glucose readings every 5 minutes. They merged the CGM’s integrated alert with the estimated hypoglycemia alert, through each of the 3 aforementioned methodologies. They used a 30-minute prediction horizon, where the methodologies scored sensitivities of 89%, 87.5%, and 89%, respectively.

Some of the prediction algorithms used in these studies used linear regressions or Kalman filters, which are computational approaches that use prior data to make short-term predictions and can also be integrated into monitoring equipment. According to the Diabetes Control and Complications Trial [24], 55% of hypoglycemic events occur during sleep; hence, some studies [12,13] addressed the issue of nocturnal hypoglycemia in T1D and argued that CGM alerts may be ineffective while the patient is sleeping [12,13].

Chase et al [12] tracked 40 patients who wore GlucoWatch CGM during the night, and they discovered that 71% of the patients did not react to the alert throughout the night. They proposed that when hypoglycemia is expected, the CGM sensor sends a signal to the pump to cease injecting insulin. To anticipate hypoglycemia, they used a mathematical model that used a system that included specific prediction algorithms. These algorithms were linear projection, Kalman filtering, hybrid infinite impulse, statistical prediction, and numerical logical algorithm. Through the use of current and prior glucose levels, these algorithms forecasted hypoglycemic events. When the number of algorithms used to forecast a hypoglycemic event exceeded the specified voting threshold, the alert was activated. Specifically, when 3 algorithms were used to prompt insulin pump suspension, nocturnal hypoglycemia was avoided, with a sensitivity of 60%. Nevertheless, using only 2 of the algorithms, nocturnal hypoglycemia occurrences were prevented with a sensitivity of 84%. Finally, this study discovered that when the voting threshold increases, the prediction rate drops, although the purpose of their proposed system was to create a balanced ratio between nocturnal hypoglycemia forecasting and the probability of false alarms.

A total of 3 prediction algorithm variants were examined in a 21-night randomized study conducted by Buckingham et al [13] using a Kalman filter–based model. The experiment comprised 19 adult patients with T1D, who were already using the MiniMed Paradigm REAL-Time insulin pump and Medtronic Sof-sensor blood glucose sensor. Pump suspension events occurred on 53% of the intervention nights using the final algorithm. Preliminary effectiveness results indicated that their final algorithm reduced nighttime hypoglycemia by approximately 50%.

### Algorithmic Inputs, Process, and Outputs

Through the increasing availability of equipment such as CGMs, insulin pumps, and physical activity trackers, along with the counting of carbohydrates by patients with T1D, a wide variety of data can be collected that can be used to predict blood glucose. Depending on the data gathered, their complexities, and the ultimate objective of the algorithm, a variety of methodologies were used in some of the studies, with 1 or 2 supplementary data inputs, which were typically the insulin doses, carbohydrates, or even both. The prementioned input data are conveniently available, as they are usually captured in sensor-enhanced pump trials and offer sufficient precision for modeling purposes. These 2 additional data inputs were processed by physiological models in many of the evaluated studies [14,15,22,23] to derive additional characteristics to determine the effects and dynamics of insulin action or meals for a better interpretation by the prediction algorithms.

There is evidence that the inclusion of insulin and carbohydrate data in prediction models often increases the performance of the algorithm, even by a very small amount. However, apart from clinical trials, in which patients are deliberately selected based on their compliance with instructions and their ability (eg, to count carbohydrates), such an input into a real-life environment seems unlikely. Table 2 presents the features that were considered and analyzed in each of the reviewed studies, and Multimedia Appendix 2 presents the number of the hypoglycemia prediction references based on the year of their considered question; it is worth noting that for 2021, we have data for the first 6 months.

**Table 2:** Features or characteristics considered in the predictive models.

Study	CGM^a^ readings	Glucose meter measurements	Insulin dosage	BMI	Carbohydrates	Meals	Activity	ECG^b^	HRV^c^	Diabetes duration	HbA_1c_^d^
Mordvanyuk et al [6]	✓		✓		✓	✓					
Paul et al [7]	✓										
Jensen et al [8]	✓		✓	✓						✓	
Zhang et al [9]		✓	✓								
Dave et al [10]	✓		✓		✓					✓	✓
Eren-Oruklu et al [11]	✓	✓									
Chase et al [12]	✓	✓		✓						✓	✓
Buckingham et al [13]	✓										✓
Georga et al [14]	✓		✓			✓	✓				✓
Bertachi et al [15]	✓		✓				✓				
Vahedi et al [16]	✓			✓			✓			✓	
Maritsch et al [17]	✓								✓		
San et al [18]		✓						✓			
Kuang et al [19]	✓										
Zhu et al [20]	✓		✓			✓					
Li, K, unpublished data, October 2019	✓		✓			✓					
Munoz-Organero et al [21]	✓		✓			✓					
Ranvier et al [22]						✓	✓	✓			
Cichosz et al [23]	✓	✓	✓					✓	✓	✓	✓

^a^CGM: continuous glucose monitoring.

^b^ECG: electrocardiogram.

^c^HRV: heart rate variability.

^d^HbA_1c_: hemoglobin A_1c_.

In a study by Georga et al [14], the authors used data from a recent patient profile to provide their support vector regression model for predicting hypoglycemia incidents during sleep, as well as in the daytime, over 30- and 60-minute time spans. With a hypoglycemia threshold of 70 mg/dL, the patient profile included glucose readings, meals, insulin dosage, and physical activity along with additional elements to account for recurrent nocturnal hypoglycemia caused by previous hypoglycemia, exercise, and sleep. Their model was developed based on a data set of 15 patients with T1D in an unrestricted environment. Nocturnal hypoglycemia predictions had a sensitivity of 94% and time delays of 5.43 and 4.57 minutes, respectively. When physical activities were not considered, the sensitivities for nonnocturnal events were 92% and 96% for the 30- and 60-minute horizons, respectively, with both time delays being <5 minutes. Nevertheless, when physical activities were considered, diurnal sensitivity was reduced by 8% and 3% in each time span. In conclusion, they suggested that their method was reliable and that both nocturnal and daytime predictions had high precision, exceeding 90%.

### Activity Wearables

Another important factor influencing blood glucose levels is physical exercise. Bertachi et al [15] examined the use of physical activity monitors to gather data on heart rate, energy expenditure, and the number of steps taken to improve the prediction ability of their model. In particular, the authors investigated the prediction of nocturnal hypoglycemia in adults with T1D through a FreeStyle Libre CGM device and a physical activity monitor (Fitbit Alta HR, Fitbit). In their 12-week study, 10 adults with T1D were examined under free-living conditions at home; details about the management of T1D, CGM, and the physical activity tracker were obtained. Supervised machine learning algorithms were applied to the data, and prediction models were developed to predict the occurrence of nocturnal hypoglycemia. The authors concluded that >70% of the nocturnal hypoglycemia could be predicted using their approach. Specifically, the prediction of the SVM model produced the highest scores, with a sensitivity of 78.75% and a specificity of 82.15%.

Overall, the inclusion of a patient activity signal as an input to the algorithm can improve its predictability, which in practice indicates that many widely available activity monitoring systems are accurate enough to be used for this task. The potential issue might be more technical in terms of merging different models and examining the variability of data formats in each system during the hypoglycemia prediction process. Other relevant information, such as stress, medical treatment, and daily events in the patient’s life, can be considered as potential inputs, which could be useful in differentiating these prediction models.

Vahedi et al [16] investigated the adaption of a machine learning–based model that predicts continuous glucose levels and aims to prevent hypoglycemia through using physiological and physical exercise data. They used the Medtronic MiniMed 530G insulin delivery device, along with the Enlite sensor, to collect 4 months of physiological measures, physical activity, and nutrition data from 93 individuals with T1D. Overall, their findings indicated that the model’s projected glucose levels were very close to the glucose values measured with the Enlite sensor.

Another machine learning model was developed in an ongoing study by Maritsch et al [17], whose objective was to identify hypoglycemia using physiological data collected from a wearable sensor. Specifically, 1 patient with T1D participated in a 1-week study, wearing an Empatica E4 smartwatch to collect physiological data and a FreeStyle Libre CGM to gather the patient’s glucose data. The reported results indicate that physiological data can indeed be used to infer hypoglycemic phases; however, frequent false-positive results were observed because of the model’s high sensitivity. However, they intend to use artificial intelligence–based techniques to make the classification output comprehensible for patients and incorporate their model into wearables to alert them about impending hypoglycemic episodes.

The ability to connect CGM, insulin pumps, and activity trackers to a mobile device can allow for the application of multiple variant algorithms and complex cloud-based estimations. One of the primary aspects in common among a few of the aforementioned prediction algorithms [6,10] is that using carbohydrate consumption, insulin dosages, and activity tracking data can improve accuracy over a forecast period. Finally, integrating several models could allow for different kinds of hypoglycemia alerts, each one designed for a certain context (activity, sleep, and type of meal).

### ECG‐Based Hypoglycemia Detection

In recent years, researchers have examined the effect of low blood glucose levels on the electrical activity of the heart. During hypoglycemia, studies revealed a lengthening of the QT interval (the time elapsed between the onset of the Q wave and the conclusion of the T wave), a rise in heart rate variability (HRV), and alterations in cardiac repolarization. Thus, monitoring ECG alterations can provide a noninvasive method for detecting the beginning of hypoglycemia. The emergence of novel ECG wearables permitted the effortless collection of cardiac signals and paved the path for hypoglycemia identification through ECG data and using deep learning techniques.

In a study by San et al [18], a deep belief network (DBN) was used to build a deep learning system for detecting the initiation of hypoglycemia based on a patient’s ECG signal. According to the authors, the probability of hypoglycemia in individuals with T1D is most affected by QT interval prolongation, although an increase in heart rate can also influence the status of the hypoglycemic event. Specifically, their suggested DBN delivers a high classification performance with feature transformation. Through the efficiency testing of the system, 15 children with T1D participated and were monitored overnight, and the findings revealed that the suggested DBN excelled and produced higher classification performance than other current methods, with a sensitivity and specificity score of 80% and 50%, respectively.

Another deep learning framework for predicting blood glucose levels was recently developed [19], which used edge inference on a microcontroller unit. The performance of the models was evaluated based on a clinical data set acquired from 12 patients with T1D whose glucose was measured with a CGM, as well as through a long short-term memory artificial recurrent neural network. Such a system could significantly aid in T1D care and eventually be used in various diabetes management wearables, such as insulin pumps and CGMs.

Generally, machine learning and deep learning approaches demonstrate significant possibilities in terms of data analysis and prediction, and they concentrate on automatically learning behaviors and extracting characteristics from large-scale data. A deep learning model was developed [20] based on a dilated recurrent neural network (DRNN) that can anticipate future glucose levels for 30 minutes. Their DRNN model acquired a considerably wider receptive field of neurons when dilation was used, with the goal of capturing long-term relationships, and they also used a transfer learning approach to take advantage of data from various patients.

A study (Li, K, unpublished data, October 2019) suggested a dual-hormone delivery approach for patients with T1D using deep reinforcement learning and based on data from the UVA-Padova T1D Simulator [25]. In terms of the hormone delivery strategy, they used double DRNNs; input data were blood glucose and carbohydrates, and output was insulin and glucagon distribution. Overall, their findings revealed that deep reinforcement learning appeared to be helpful in developing customized hormone delivery strategies for patients with T1D.

In another deep learning–based hybrid model [21], the authors attempted to imitate the metabolic behavior of physiological blood glucose techniques based on both computer-generated and actual patient data. Furthermore, they simulated a set of differential equations for insulin and carbohydrate intake through a long short-term memory recurrent neural network. Results demonstrated that their model performs better for simulated patients because of the intricacy of the insulin and carbohydrate intake dependence on blood glucose levels, which is restricted to a specific cluster of parameters.

In a noninvasive approach, Ranvier et al [22] aimed to detect hypoglycemic events based on the continuous collection of sensed data from an off-the-shelf sensor belt; the authors based their method on 2 distinct models. The first one leveraged a physiological consequence of hypoglycemia, namely, an alteration of the user ECG’s features. They additionally used the accelerometer and breathing sensor of the belt to infer the energy expenditure of the patient with T1D and correlated it with the food intake to estimate the blood glucose level. They then combined these 2 models to improve the accuracy of their prediction.

Cichosz et al [23] proposed a novel algorithm for hypoglycemia prediction, where they obtained data from 10 patients with T1D, who were observed during insulin-induced hypoglycemia, and the collected blood glucose samples were used as a reference. Their equipment involved the calculation of ECG, lead II, and a Minimed Guardian RT CGM, which generated a reading every 5 minutes. The extracted HRV patterns were incorporated into a mathematical prediction algorithm along with the CGM data. Cichosz et al [23] treated early prediction as a pattern recognition problem based on a fixed hypoglycemia level (3.9 mmol/L). Thus, measuring blood glucose from each patient was used as a reference to categorize each 5-minute reading into 2 groups: in healthy range blood glucose (Cn) or hypoglycemia (Chy). Features obtained from HRV and CGM before each blood glucose measurement were used to assess if that time point was below the hypoglycemic threshold of 3.9 mmol/L. As a result, a total of 903 samples were evaluated using the proposed algorithm, with a sensitivity of 79% and an accuracy of 99%. The algorithm was able to predict all 16 hypoglycemic events with no false positives and had a lead time of 22 minutes relative to the CGM device.

These studies indicate that ECG could be used in a free-living environment to assist patients in detecting hypoglycemic episodes. Upgraded equipment and optimized algorithms could make certain methods more precise and simpler to deploy in practice. Although patients with T1D might not be the first to benefit from these technological approaches, other non-T1D patients experiencing hypoglycemic episodes arising from other conditions, such as endocrine, hepatic, or cardiac disorders, could be positively affected by these ECG-based algorithms.

## Discussion

### Principal Findings

In the context of T1D hypoglycemia risk management, several hypoglycemia or blood glucose level prediction approaches were assessed in this review. Each of these approaches included different techniques and tools that were used for blood glucose level prediction. In general, hypoglycemia prediction algorithms can offer a valuable alternative to patients with T1D to prevent possible episodes, as there are many patients that experience asymptomatic hypoglycemic episodes.

Several of the approaches reviewed have already been incorporated into commercially available systems; that is, the approach proposed by Bertachi et al [15] using a FreeStyle Libre CGM device and a Fitbit Alta HR physical activity monitor, which has been shown to effectively decrease hypoglycemic episodes. A common key aspect of several of the evaluated studies is that the inclusion of carbohydrate consumption data, insulin dosages, or exercise data can enhance the accuracy of the algorithm in the context of a defined (medium- or long-term) forecast horizon. Furthermore, integrating various models could allow for several stages of hypoglycemia alerts, each of which could be tailored to a unique scenario, such as a postmeal, postactivity, or during sleep prediction [26].

Unfortunately, there can be significant variations in accuracy when predicting blood glucose levels. Data collection in these types of studies can be affected by a variety of limiting factors, including inefficient hardware, constrained health care settings, patient noncompliance with research procedures, and barriers because of extensive biomedical data records. These impediments force machine learning researchers to cope with flawed data and seek workarounds for their prediction models [27]. Furthermore, the prediction accuracy highly depends on the type of diabetes, the patient’s lifestyle [28], and the existence of any other chronic disease. Some underlying mechanisms, such as age, gender, intestinal microbiota, psychological factors, and genetic traits, may also contribute to variations in outcomes [29]. In addition, we noticed that many of the previously mentioned methodologies were trained on computer-generated patients from simulators (Li, K, unpublished data, October 2019) [6,20,21] or on relatively restricted data sets involving strongly competent patients [7,17,22]. These patients strictly follow the given research guidelines or are in a monitoring environment, which abstains from everyday life where patients mostly do not monitor events, such as heart rate, regularly, which are usually essential for these methodologies. We also noticed that several methodologies used a limited number of features [7,9,11,13,18,19]. This can have a significant impact on the final results, as several factors can affect blood glucose levels, each with different severity. In contrast, some studies used a wide variety of data, such as the approach proposed by Cichosz et al [23], in which 7 different types of features were included. Specifically, they used CGM readings, glucose meter measurements, insulin dosage, ECG, HRV, diabetes duration, and hemoglobin A_1c_ levels and achieved an accuracy of 99% and a sensitivity of 79%. In our opinion, to improve the overall efficiency of these approaches, it is necessary for researchers to obtain larger data sets and take into consideration a higher number of features in their approaches. A gold standard data set for glucose level prediction in patients with T1D would assist data analysts in experimenting, comparing, and fine-tuning their models accordingly.

CGM sensors are considered a revolution in diabetes treatment [30], are expected to enhance data-driven strategies for personalized diabetes therapy, and can provide real-time data for the creation of predictive models [31]. Clinical studies of such algorithms are projected to increase in the future as prediction approaches are integrated into CGM systems and other devices. Furthermore, the evolution of deep learning algorithms trained using streaming data provides promising results for glucose prediction [20]. The first priority for a hypoglycemia prediction model is to alert the patient before hypoglycemia occurs. Researchers attempted to predict hypoglycemic episodes at various prediction horizons in the cited studies, varying from 0 to 60 minutes. Altogether, the advantages for patients with T1D are evident, as they are empowered to make preventive decisions before their blood glucose levels reach critical points [32]. As with any new equipment, education is required to avoid the negative side effects of overreactions.

Nevertheless, the current CGM technology has drawbacks such as limited life span, skin irritation, adhesive problems, and consumable expenses, which may make it unaffordable for lifelong tracking and prediction. The challenge is to use mainstream noninvasive sensors such as wristbands and smartwatches to build reliable predictive models for hypo- and hyperglycemia following the paradigm of ECG and HR sensors available in mainstream devices and used to assist people with cardiac conditions [33].

### Limitations

This review should be interpreted within the context of its limitations. We used a limited set of terms for the search of the literature. Keywords for specific algorithms were not used and we might have inadvertently omitted studies that could have contributed to the progress made in algorithms for T1D hypoglycemia prediction. We searched for articles in a limited number of databases (ie, *PubMed*, *Google Scholar*, *IEEE Xplore*, and *ACM* Digital Library), which represent the most widely used databases internationally. We did not hand search any studies reported in other reviews or the included studies, and we did not assess the interrater reliability. On the basis of our inclusion and exclusion criteria, a small number of eligible studies was included and examined in this review, which limits the generalizability of the findings.

### Conclusions

In this systematic review, we included a wide range of hypoglycemia prediction algorithms and systems, some of which used specific medical or activity devices, such as CGMs and activity trackers. Nevertheless, these approaches cannot be recommended to patients on their own; they must be supported by a comprehensive plan to be effective in supporting medical care. Specifically, before deploying the right equipment or technology to aid a patient with T1D, education and medication management are required to decrease the probability of developing hypoglycemia. Overall, we conclude that other approaches to hypoglycemia prediction will be challenged compared with the commonly used CGMs in the following years, as they are restricted to event detection, and CGMs also have the potential to notify patients about their blood glucose variability.

## Appendix

Multimedia Appendix 1 Number of algorithms used by studies categorized based on similarity.

Multimedia Appendix 2 The number of references based on the year of their considered question.

## Abbreviations

CGM: continuous glucose monitoring
DBN: deep belief network
DRNN: dilated recurrent neural network
ECG: electrocardiogram
GARCH: generalized autoregressive conditional heteroscedasticity
HRV: heart rate variability
PRISMA: Preferred Reporting Items for Systematic Reviews and Meta-Analyses
SVM: support vector machine
T1D: type 1 diabetes
