# Supplementary material for: Type 1 Diabetes Hypoglycemia Prediction Algorithms: Systematic Review
Source: JMIR Diabetes. 2022 Jul 21;7(3):e34699. doi: 10.2196/34699 (PMC9353679; doi:10.2196/34699)
Supplement: Multimedia Appendix 1 [file diabetes_v7i3e34699_app1.docx]

Multimedia Appendix 1. Number of algorithms used by studies categorized based on similarity.

| Algorithms categorized based on similarity | Number of studies that used the algorithm |
| --- | --- |
| Artificial neural network | 12 |
| Regression | 7 |
| Decision tree | 4 |
| Deep learning | 5 |
| Probabilistic or statistical dependence | 2 |
| Supervised machine learning | 4 |
