# Supplementary material for: Type 1 Diabetes Hypoglycemia Prediction Algorithms: Systematic Review
Source: JMIR Diabetes. 2022 Jul 21;7(3):e34699. doi: 10.2196/34699 (PMC9353679; doi:10.2196/34699)
Supplement: Multimedia Appendix 2 [file diabetes_v7i3e34699_app2.docx]

Multimedia Appendix 2. The number of references based on the year of their considered question.

| Number of references | The year of their considered question |
| --- | --- |
| 1 | 2008 |
| 2 | 2010 |
| 3 | 2013 |
| 1 | 2014 |
| 1 | 2015 |
| 3 | 2016 |
| 1 | 2017 |
| 2 | 2018 |
| 1 | 2019 |
| 4 | 2020 |
| 1 | 2021 |
